# Olfactory Training and Visual Stimulation Assisted by a Web Application for Patients With Persistent Olfactory Dysfunction After SARS-CoV-2 Infection: Observational Study

**DOI:** 10.2196/29583

**Published:** 2021-05-27

**Authors:** Fabrice Denis, Anne-Lise Septans, Lea Periers, Jean-Michel Maillard, Florian Legoff, Hirac Gurden, Sylvain Moriniere

**Affiliations:** 1 Institut Inter-Regional Jean Bernard - ELSAN Le Mans France; 2 Weprom Angers France; 3 Service d'Otorhinolaryngologie Centre Hospitalier Universitaire Bretonneau Tours France; 4 anosmie.org Association Alencon France; 5 Kelindi Lille France; 6 Unite de Biologie Fonctionnelle Adaptative, Unite Mixte de Recherche 8251 Centre National de Recherche Scientifique, Université de Paris Paris France

**Keywords:** olfactory dysfunction, SARS-CoV-2, olfactory training, web application, eHealth, real-life study, COVID-19, app, training, stimulation, olfactory, dysfunction, smell, observational, senses, nose

## Abstract

**Background:**

Persistent olfactory dysfunction is a significant complication of SARS-CoV-2 infection. Olfactory training involving aromatic oils has been recommended to improve olfactory recovery, but quantitative data are missing.

**Objective:**

We aimed to quantify the benefit of olfactory training and visual stimulation assisted by a dedicated web application for patients who experienced olfactory dysfunction for ≥1 month.

**Methods:**

We performed an observational, real-life, data-based study on a cohort of patients who experienced at least 1 month of persistent olfactory dysfunction between January 30 and March 26, 2021. An analysis was performed after a mean olfactory training time of 4 weeks, and at least 500 patients were assessable for primary outcome assessment. Participants exposed themselves twice daily to odors from 4 high-concentration oils and visual stimulation assisted by a dedicated web application. Improvement was defined as a 2-point increase on a 10-point, self-assessed olfactory visual analogue scale.

**Results:**

In total, 548 patients were assessable for primary outcome assessment. The mean baseline, self-assessed olfactory score was 1.9 (SD 1.7), and this increased to 4.6 (SD 2.8) after a mean olfactory training time of 27.7 days (SD 17.2). Olfactory training was associated with at least a 2-point increase in 64.2% (352/548) of patients. The rate of patients’ olfactory improvement was higher for patients who trained for more than 28 days than that rate for patients who trained for less than 28 days (73.3% vs 59%; *P*=.002). The time to olfactory improvement was 8 days faster for patients with hyposmia compared to the time to improvement for patients with anosmia (*P<.*001). This benefit was observed regardless of the duration of the olfactory dysfunction.

**Conclusions:**

Olfactory training and visual stimulation assisted by a dedicated web application was associated with significant improvement in olfaction, especially after 28 days of olfactory training.

## Introduction

Anosmia is a frequent symptom of SARS-CoV-2 infection, and its duration is usually less than 2 weeks before recovery [1-3]. However, at least 10% of patients with SARS-CoV-2 infection will experience persistent and chronic olfactory dysfunction such as diminished smell (hyposmia) or the loss of smell (anosmia), which have been shown to result in a decreased quality of life, depressive symptoms, and nutrition issues [4-6]. One treatment option that is recommended for persistent olfactory dysfunction is daily olfactory training involving high-concentration aromatic oils [7]. This showed significant results in treating postinfectious olfactory loss in a randomized, controlled, multicenter study [8]. In this trial, after 18 weeks of olfactory training, olfactory function improved in 63% of patients who experienced olfactory dysfunction for a duration of less than 12 months and used high-concentration oils, whereas olfactory function improved in 19% of patients in the control group who used low-concentration oils. Moreover, the combination of visual stimulations and olfactory training may improve recovery results [9].

No data about olfactory training for persistent olfactory dysfunction are available on patients with SARS-CoV-2 infection and persistent olfactory dysfunction, but most patients who experience hyposmia or anosmia for 30 days or more seem to have a low rate of spontaneous recovery [4].

In order to quantitatively study the time course of olfactory scores during olfactory training in real life, we developed a web application dedicated to olfactory training and visual stimulations as well as the self-assessment and follow-up of olfactory scores. We assessed the results in a real-life observational study.

## Methods

The web application users were recruited via a national media campaign in France that was disseminated through social media, radio, and magazines between January 30 and February 15, 2021.

This observational, data-based study was approved by the French National Health Data Institute, which reviews the ethical conduct of human subjects research, data confidentiality, and safety. To participate, individuals were required to connect to the free covidanosmia.eu web application and provide electronic agreement. Respondents anonymously self-entered sociodemographic data and real-time polymerase chain reaction test results and confirmed a diagnosis of SARS-CoV-2–related olfactory dysfunctions. Patients were also asked to complete items about comorbidities, the duration of olfactory symptoms, and the self-assessed intensity of olfactory dysfunction by providing subjective ratings with a visual analogue scale of 0 (no smell) to 10 (no smell alteration) [10]. Patients were retained in the study analysis if they were diagnosed with SARS-CoV-2–related olfactory dysfunction that persisted for at least 1 month and reported at least 7 days of olfactory training, and if their last olfactory function assessment on the web application diary was available. The exclusion criteria were normosmia (visual scale score of >7); other causes of olfactory dysfunction such as chronic rhinosinusitis, nasal polyposis, allergic or idiopathic rhinitis, posttraumatic olfactory loss, and other acute or chronic nasal diseases (eg, acute viral infections); malignant tumors or oncology therapies (radiation therapy and chemotherapy), and a history of surgery for the nose or paranasal sinuses.

Patients had to obtain the olfactory training kit from the web application or from their pharmacist. Olfactory training was performed for a maximum period of 16 weeks. The web application provides videos, tutorials for the training, and periodic encouragements. Participants exposed themselves twice daily to odors from the following four high-concentration oils: phenyl ethyl alcohol (rose odor from *Geranium rosa*), eucalyptol (eucalyptus odor), citronellal (lemon odor), and eugenol (cloves odor). These four odorants were chosen to represent the primary odor categories created by Henning [11,12]. Participants sniffed each odor for approximately 15 seconds while blinded and repeated this process 30 seconds later once while the name and picture of the oil component was on the screen of the web application (eg, a picture of a lemon during the lemon oil sniffing process). Patients were asked to train in the morning and in the evening, resulting in a total of 4 exposures per day per odor. They were asked to keep a daily diary on the web application, in which they rated their overall olfactory abilities for each oil with subjective ratings on a visual analogue scale.

We assessed the rate of self-assessed improvement in overall olfactory function along with training times by using data that were collected anonymously from the web application diaries of patients. Improvement was defined as an increase of ≥2 points on the olfactory visual analogue scale. The analysis was performed when the mean olfactory training time of the study population was at least 4 weeks and when at least 500 patients were assessable for primary outcome assessment.

Categorical variables were summarized by using frequencies and percentages, and chi-square tests or Fisher exact tests were used to make comparisons. For quantitative variables, which were summarized with descriptive statistics, the following values were presented: N values, means, and SDs. A *t* test was used to compare groups, and the analysis of variance test was used for comparisons of more than 2 groups.

The Kaplan-Meier methodology was used to summarize time-to-event variables. Plots of Kaplan-Meier product limit estimates for time-to-event variables were drawn, and medians were presented in addition to CIs set at 95%. To compare Kaplan-Meier curves of the two groups, the log-rank test was used.

The level of statistical significance was 5% for all statistical tests (exploratory tests). To analyze predictive factors of assessment, logistic regression was used in order to calculate odds ratios, which were presented with CIs set at 95%.

All statistical analyses were conducted with SAS (Statistical Analysis System), version 9.3 (SAS Institute Incorporated).

## Results

**Figure 1:**
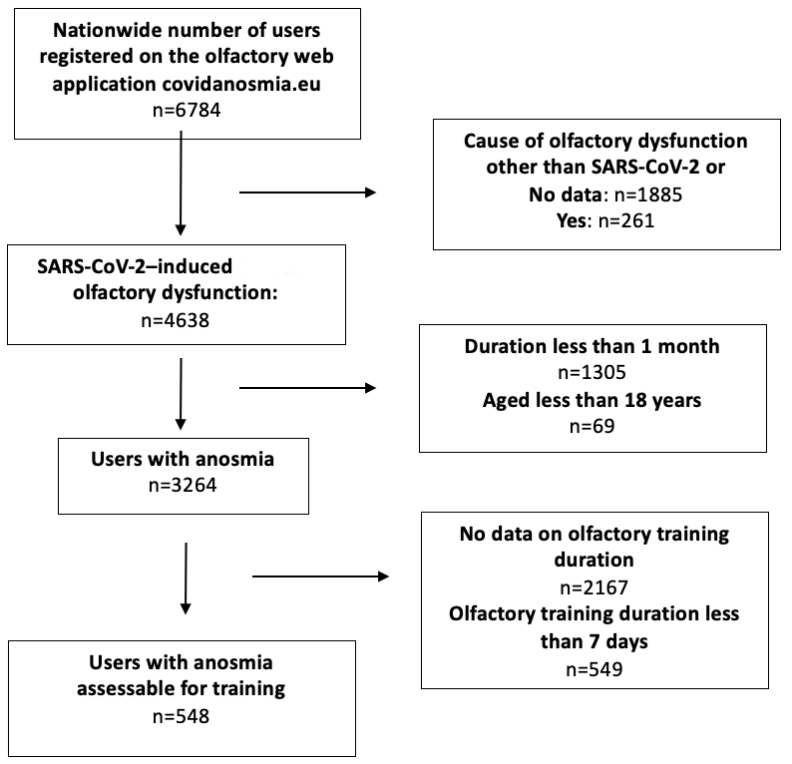
Flowchart of patients who used a web application for olfactory training.

Between January 30 and March 26, 2021, the web application was used by 6755 unique individuals who completed the baseline questionnaires. Of these individuals, 548 met the inclusion criteria and were assessable for outcome assessments (Figure 1).

The 548 assessable patients’ median age was 42 years (range 18-84 years). Of these patients, 65.5% (n=359) were female, 32.1% (n=176) estimated having a smell sense that was more developed than average before olfactory dysfunction, 69.3% (n=380) reported that smell sense had an important role in their life, and 30.7% (n=168) did not care about their smell sense before SARS-CoV-2 infection. Of the 548 assessable patients, 111 (20.3%) experienced anosmia (level 0 on the olfactory scale), 287 (52.4%) experienced severe hyposmia (level 1 or 2 on the olfactory scale), 125 (22.8%) experienced moderate hyposmia (levels 3-5 on the olfactory scale), 25 (4.6%) experienced mild hyposmia (levels 6-7), 279 (50.9%) reported a reduction in or loss of taste, and 289 (52.7%) reported parosmia. Patients’ baseline characteristics are shown in Table 1. The mean baseline olfactory function of users who were registered on the web application but underwent less than 7 days olfactory training or did not record their last olfactory function assessment on the web application diary (n=2824; olfactory function score: mean 2.23) was higher than that of the studied population (n=548 patients; olfactory function score: mean 1.9; Student test *P*<.001).

The mean baseline, self-assessed olfactory score was 1.9 (SD 1.7), and this increased to 4.6 (SD 2.8) after a mean olfactory training time of 27.7 days (SD 17.2 days; range 7-65 days).

Olfactory training was associated with at least a 1-point increase on the olfactory scale in 82.1% (450/548) of patients, at least a 2-point increase (ie, the primary outcome) in 64.2% (352/548) of patients, and at least a 3-point increase in 49.3% (270/548) of patients during the study period. The rate of olfactory improvement in patients who experienced anterior olfactory dysfunction for 12 months was 58.3%. With regard to patients whose olfactory function score increased by at least 2 points, their scores increased by a mean of 4.1 points (SD 1.9 points).

**Table 1:** Patients’ characteristics.

Variables		Value, n (%)	
**Sex**			
	Male		189 (34.5)
	Female		359 (65.5)
**Smell level before smell dysfunction**			
	Standard		354 (64.6)
	Less developed than average		18 (3.3)
	More developed than average		176 (32.1)
**Role of smell before smell loss**			
	Did not care about smell		168 (30.7)
	Important role		380 (69.3)
**Smell level at baseline**			
	0		111 (20.3)
	1-2		287 (52.4)
	3-5		125 (22.8)
	6-7		25 (4.6)
**Olfactory dysfunction duration (months)**			
	1-2		61 (11.1)
	2.1-3		250 (45.6)
	3.1-6		167 (30.5)
	6.1 to ≥12		70 (12.8)
**Taste dysfunction**			
	None		269 (49.1)
	Dysfunction		279 (50.9)
**Parosmia**			
	No		259 (47.3)
	Yes		289 (52.7)

**Figure 2:**
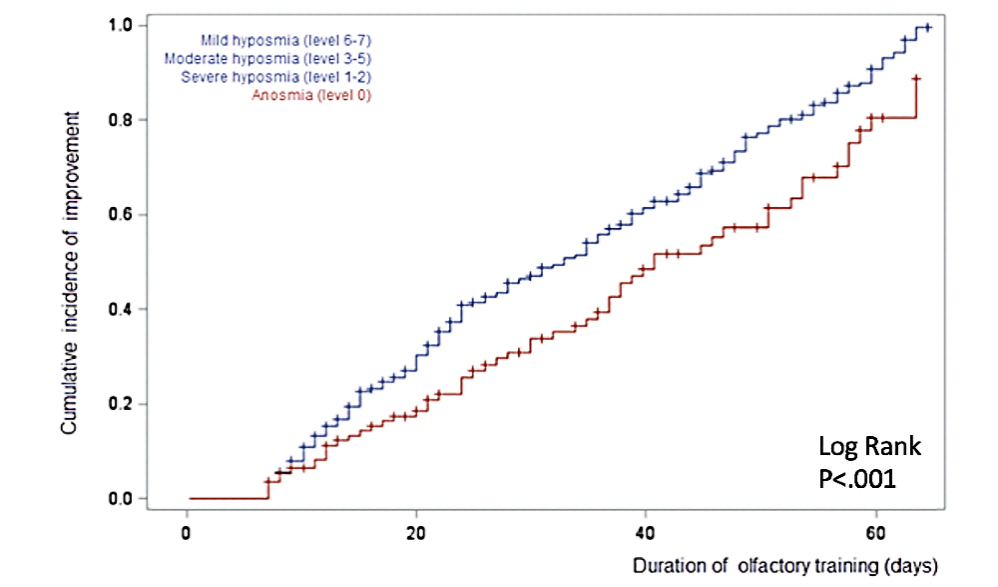
Cumulative incidence of olfactory improvement according to the olfactory training durations of patients with anosmia and hyposmia. The data of patients with mild, moderate, and severe hyposmia were pooled in the blue curve.

The duration of the training was associated with better outcomes, and the time to olfactory function improvement was longer in patients with anosmia (olfactory training duration for a 50% probability of improvement: mean 41 days; range 36-53 days) than in patients with hyposmia (mean 33 days; range 28-36 days; log-rank *P*<.001; Figure 2). There were no significant differences in the duration of training among patients with severe, moderate, and mild hyposmia (severe vs moderate *P*=.052; severe vs mild *P*=.96 and moderate vs mild *P*=.87).

The rate of patients’ olfactory improvement (at least a 2-point increase on the olfactory scale) was higher for patients who trained for more than 28 days than that rate for patients who trained for less than 28 days (73.3% vs 59%; *P*=.002). Patients who underwent 28 days of olfactory training or more and benefited from olfactory improvement exhibited a mean improvement of 4.4 points (SD 2.0 points) on the olfactory scale, whereas a mean improvement of 3.8 points (SD 1.8) was observed in patients who underwent less than 28 days olfactory training (Student test *P*=.01).

**Figure 3:**
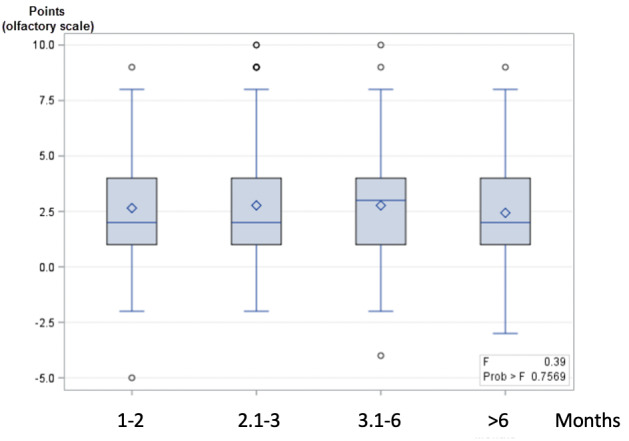
Mean improvement of olfactory function stratified by the duration of persistent olfactory dysfunction. Improvement was assessed with a self-assessed olfactory scale of 0-10 after olfactory training.

The mean improvement in self-assessed olfactory scale scores was similar regardless of the anteriority of the olfactory dysfunction (*P*=.70; Figure 3). No other predictive factors were highlighted (Table 2).

**Table 2:** Logistic regression analysis for determining the predictive factors of olfactory function improvement (ie, an increase of ≥2 points on the olfactory scale).

Variables			Univariate analysis		
			Hazard ratio (95% CI)		*P* value
**Gender**					.77
	Male	1 (N/A^a^)			
	Female	0.945 (0.654-1.366)			
Age			1.012 (0.997-1.026)		.11
**COVID-19 tests**					>.99
	Positive test	1 (N/A)			
	No test	0.934 (0.220-3.955)			
	Yes; negative test	1.058 (0.462-2.425)			
	Yes; no test	1.074 (0.521-2.212)			
**Anosmia duration (months)**					.85
	>6	1 (N/A)			
	1	1.287 (0.627-2.643)			
	2	1.078 (0.625-1.861)			
	3-6	1.212 (0.680-2.160)			
**Smell importance**					.65
	Standard	1 (N/A)			
	Less than standard	0.666 (0.256-1.730)			
	Better than standard	0.909 (0.624-1.325)			
**Taste lost**					.50
	No	1 (N/A)			
	Yes	1.128 (0.795-1.600)			
**Parosmia**					.44
	No	1 (N/A)			
	Yes	1.149 (0.810-1.630)			
**Training duration (days)**					.002
	<28	1 (N/A)			
	≥28	1.802 (1.247-2.604)			

^a^N/A: not applicable.

After a mean olfactory training time of 28 days, we observed that 17.1% (94/548) of patients had an olfactory score of ≥8. This high recovery was observed in patients regardless of the anteriority of the olfactory dysfunction (*P*=.93); 43.6% (41/94) of these patients experienced more than 3 months of olfactory dysfunction whereas 56.4% (53/94) experienced less than 3 months of dysfunction. However, patients with an olfactory training score of ≥8 had significantly higher baseline scores than users who did not achieve this score after training; the mean baseline scores were 2.9 (SD 2.0) for patients who achieved high recovery and 1.6 (SD 1.5) for those who achieved lower recovery (*P*<.001).

## Discussion

This study is the first to prospectively assess the real-life benefit of olfactory training for patients who experience persistent olfactory dysfunction after SARS-CoV-2 infection. The mean duration of training was 28 days. In our cohort of 548 patients who underwent olfactory training assisted by the web application, an improvement of 2 points or more in a subjective self-assessed olfactory scale was reported by 64.2% (352/548) of patients. Beyond 28 days of training, the rate of improvement was significantly higher than the rate of improvement after <28 days of training (72.2% vs 59%; *P*=.002). The time to olfactory improvement was 8 days longer for patients with anosmia compared to the time to improvement for patients with hyposmia. Improvement was observed regardless of the anteriority of the olfactory dysfunction. High recovery, that is, normal or subnormal self-assessed olfactory function, was observed in 17% (93/548) of patients regardless of the anteriority of the olfactory dysfunction, but high recovery occurred more frequently in patients with higher baseline olfactory scores (mean 2.9).

Our data are in line with a previous randomized trial on postinfectious olfactory loss [8]. In this trial, after 18 weeks of olfactory training, olfactory function improved in 63% of patients who experienced olfactory dysfunction for a duration of less than 12 months and used high-concentration oils, whereas olfactory function improved in 19% of patients in the control group who used low-concentration oils. We used high-concentration oils in combination—the same 4 odorants used by Damm et al [8]—in a 2-step process for each oil. The first step involved blind olfactory stimulation with a given oil like in the Damm et al [8] trial. The second step, which followed the first step and involved the same oil, was enriched via visual stimulation with a picture of the oil component on a smartphone screen, which was delivered by the web application. We chose this new approach to reinforce the olfactory trial with a mixed olfactory-visual trial, as some previous data have suggested that human olfactory perception can substantially benefit from visual cues. This suggests that there is important cross-modal integration between olfactory and visual modalities [9,12,13]. An ongoing, 4-arm, randomized trial is assessing the best modalities of training to improve olfactory training results [14]. The use of a web application is a promising method for improving olfactory training because it allows for visual stimulation, visual tutorials, the provision of encouragements, and results monitoring. Web applications have been shown to be useful during the SARS-CoV-2 pandemic for triaging patients and assessing trends of the outbreak at a large scale [15-17].

Our patients experienced persistent anosmia for 2 to 12 months, and the rapid recovery that was observed regardless of the anteriority of the anosmia suggests that olfactory improvement was a direct effect of the training. Postinfectious olfactory dysfunction that is not caused by SARS-CoV-2 is associated with moderate rates of spontaneous recovery. Hendriks [18] reported that spontaneous recovery occurs in 35% of patients over a period of approximately 12 months. In a retrospective series of 262 subjects with a mean follow-up time of 14 months, Reden et al [19] reported a 32% improvement in olfactory function, which was assessed with the objective “Sniffin’ Sticks” test, and an increase of at least 6 points in threshold discrimination identification scores. Hummel et al [7] reported a short-term recovery rate of 6% to 8% within 4 months and used the same olfactory tests and definitions for improvement as those of Reden et al [19]. More recently, Havervall et al [20] reported that the incidence rate of olfactory dysfunction after mild SARS-CoV-2 infection among seropositive health care workers was 14.6%, 10.8%, and 9% at 2, 4, and 8 months after infection, respectively, meaning that the spontaneous recovery rate is low [20].

Spontaneous recovery after persistent olfactory dysfunction in patients with SARS-CoV-2 infection is not well described. Vaira et al [4] reported a mean score of 1 on a 10-point analogue subjective olfactory scale (the same one we used) between 30 and 60 days for 138 patients who did not undergo olfactory training, and 20% of patients exhibited olfactory improvement. Our data suggest that improvement can be achieved tardily after 2 months of training. In our study, olfactory training and visual stimulation assisted by a dedicated web application were associated with 73.3% (165/225) of patients whose olfactory function improved by 2 points or more after at least 28 days olfactory training and a mean improvement of 4.4 points [4]. In another study, Lechien et al [21] reported that 15.3% of patients with anosmia and 4.7% of patients with hyposmia did not objectively recover olfaction after 60 days and 6 months, respectively. The comparison of our study with other studies is, however, limited because different olfactory tests and scale evaluations were used [21].

Our study had several limits. There were many excluded patients. Selection bias may exist because we believe that patients who do not feel improvement will more readily stop undergoing training. This could be due to confusion about how benefits are statistically better if patients follow the training regimen for more than 28 days. The mean baseline olfactory function of users who were registered on the web application but underwent less than 7 days of olfactory training or did not record their last olfactory function assessment on web application diary (n=2824; olfactory function score: mean 2.23) was higher than that of the studied population (n=548; olfactory function score: mean 1.9; Student test *P*<.001). The distribution of the olfactory dysfunction severity among patients suggests that patients with more severe olfactory dysfunction from the whole population were retained in the analysis. These data suggest that the results of olfactory training could be better in the whole population than those in the studied population.

There was no control group in this study; therefore, it remains unclear whether the incidence of spontaneous recovery distorted the results. The scale that was used to measure olfactory dysfunction and changes was subjective, as it was a self-assessment analogue scale; scores were self-reported and data about olfactory assessment were not confirmed by physicians and objective tests. However, the possibility of conducting olfactory training at home increased the number of recruited patients and resulted in higher levels of olfactory function recovery compared to those of spontaneous improvement.

Olfactory training and visual stimulation assisted by a dedicated web application was associated with significant olfactive improvement in persistent olfactory dysfunction following SARS-CoV-2 infection, especially after 28 days of olfactory training.

## Abbreviations

SAS: Statistical Analysis System
