# Correction: Olfactory Training and Visual Stimulation Assisted by a Web Application for Patients With Persistent Olfactory Dysfunction After SARS-CoV-2 Infection: Observational Study

**DOI:** 10.2196/32120

**Published:** 2021-07-16

**Authors:** Fabrice Denis, Anne-Lise Septans, Lea Periers, Jean-Michel Maillard, Florian Legoff, Hirac Gurden, Sylvain Moriniere

**Affiliations:** 1 Institut Inter-Regional Jean Bernard - ELSAN Le Mans France; 2 Weprom Angers France; 3 Service d'Otorhinolaryngologie Centre Hospitalier Universitaire Bretonneau Tours France; 4 anosmie.org Association Alencon France; 5 Kelindi Lille France; 6 Unite de Biologie Fonctionnelle Adaptative, Unite Mixte de Recherche 8251 Centre National de Recherche Scientifique, Université de Paris Paris France

In “Olfactory Training and Visual Stimulation Assisted by a Web Application for Patients With Persistent Olfactory Dysfunction After SARS-CoV-2 Infection: Observational Study” (J Med Internet Res 2021;23(5):e29583), one error was noted.

In the originally published article, the “Edited by” credit was listed incorrectly. This has been corrected from “Edited by C Basch, G Eysenbach” to “Edited by G Eysenbach”.

The correction will appear in the online version of the paper on the JMIR Publications website on July 16, 2021, together with the publication of this correction notice. Because this was made after submission to PubMed, PubMed Central, and other full-text repositories, the corrected article has also been resubmitted to those repositories.

